# Growing Medical Educators from Medical Students by Supporting Self-directing Learning Opportunities Born Out of Cadaveric Dissection

**DOI:** 10.1007/s40670-023-01968-9

**Published:** 2024-02-20

**Authors:** Ian Kuo, Bradley Hunt, David Doyle, Patrick Fakhoury, Hyungee Ha, Lucas Garmo, Marian Cardwell, Aleah Gillenkirk, Emily Ridge, Tom Ren, Nicholas Sirhan, Nicole Ireland, Taylor Fegan, Katherine McCollum, Kiara Timmerman, Kyle Root, Zachary S. Klukkert, Jyotsna Pandey

**Affiliations:** 1 Central Michigan University College of Medicine, 1280 East Campus Drive, Mount Pleasant, MI 48859 USA; 2 Department of Anatomy and Cell Biology, Oklahoma State University Center for Health Sciences, 1111 West 17th Street, Tulsa, OK 74107 USA

**Keywords:** Self-directed learning, Medical education, Anatomy dissection, Cadaver pathology, Medical educator

## Abstract

This study focuses on a subset of medical students who participated in an anatomy dissection program and undertook an additional self-directed learning (SDL) project investigating incidental findings of cadaveric pathology. The value of SDL activity is explored as a means of enhancing medical student education, particularly its student perceived value in preparing and developing them as future medical educators. It was assessed whether the project advanced student interest in medical education by analyzing their motivations for participation. The results of the study highlight the potential of SDL as an experiential learning opportunity for medical students and the role of anatomic pathology in connecting multiple domains of medical education.

## Introduction

Through the study of anatomic pathology and histological specimens during cadaveric dissection, medical students may acquire a deeper understanding of typical and aberrant tissue structure, thereby connecting their training to diverse domains such as anatomy and pathology [1, 2]. This knowledge not only provides scaffolding for the diagnosis and treatment of various medical conditions but also allows for development into future medical educators, enhancing their ability to connect pathological manifestations with their histological origins. Current educational practices have placed less emphasis on traditional didactic modalities such as laboratory-based histological investigation, leading to lower academic performance in the subject of histology [3] especially when connecting it to clinical features and gross pathology. However, such training can provide a blending of clinically relevant learning [1, 4, 5]. It has been shown that well-designed anatomic pathology-based training programs can enhance subject knowledge and foster a deeper appreciation for their practical application in healthcare [6, 7]. Collectively, an approach like this promotes understanding and appreciation of the importance of anatomic pathology across multiple scales, bridging clinically relevant domains, by integrating gross pathology, histology, pathophysiology, and anatomy. However, available literature does not expand on if self-directed learning (SDL) activities [8] combined with such anatomy–pathology blended programs can provide students with the necessary training to develop their potential as future medical educators.

Central Michigan University College of Medicine offers a Summer Anatomy Dissection program for rising second-year medical students. In this extracurricular program, they perform dissections of cadavers to enhance their anatomical knowledge and to assist in preparing cadaveric specimens and teaching materials to benefit their peers in the preclinical curriculum. A subset of these students in this program investigated incidental findings during dissection via additional means, i.e., independent research and collaboration with faculty. The present study aims to characterize the motivation behind students’ pursuit of this SDL activity to assess whether it advanced the students’ interest in medical education or development as a medical educator.

## Methods

The overarching goal of the anatomy dissection program is to engage second-year medical students in the preparation of anatomical cadaveric prosections which are later used to educate first- and second-year medical students. Students self-identify their individual learning objectives for the program at the start of the program, including bolstering their understanding of human anatomy, developing familiarity with surgical instruments, practicing collaboration with their peers, research project development, and/or developing an educational project and practicing teaching. As part of the dissection program, students are provided with a limited history with age of the donor and cause of death.

A subset of 14 students (60% of participants) in the summer program developed individual additional projects outside of the planned programming to investigate gross pathologies as per cause of death or those that were incidentally detected. They initiated a voluntary, faculty-supported, research-based correlation of their findings to clinical manifestations of the disease or disorder, pathophysiologic changes, and study of anatomic and histopathologic changes of the anomaly or disorder. These investigations were self-directed and included independent research for better understanding of the disease or disorder. The students collaborated for guidance with pathology and anatomy faculty, as needed. In this process, the students received additional training in histological processing techniques, namely, the excision of the tissue of interest, and subsequent embedding, slicing, staining, imaging, and identification.

Following the SDL activity, the students’ motivation to participate in the activity was assessed, as was their own perceptions of their growth as medical educators resulting from the activity. Student responses to the following Qualtrics-hosted survey were collected:

1. The Summer Anatomy Dissection program is designed as an educational activity where students prepare cadavers for in-class teaching and learning activities. You found a cadaveric pathology during this activity. What was your motivation for engaging to investigate pathology further as a faculty-guided self-directed activity?
2. The Accreditation Council for Graduate Medical Education (ACGME) emphasizes the value of training “residents as teachers.” Do you feel that your voluntary pursuit of this self-directed learning activity advanced your preparation to be a future medical educator?

**Fig. 1:**
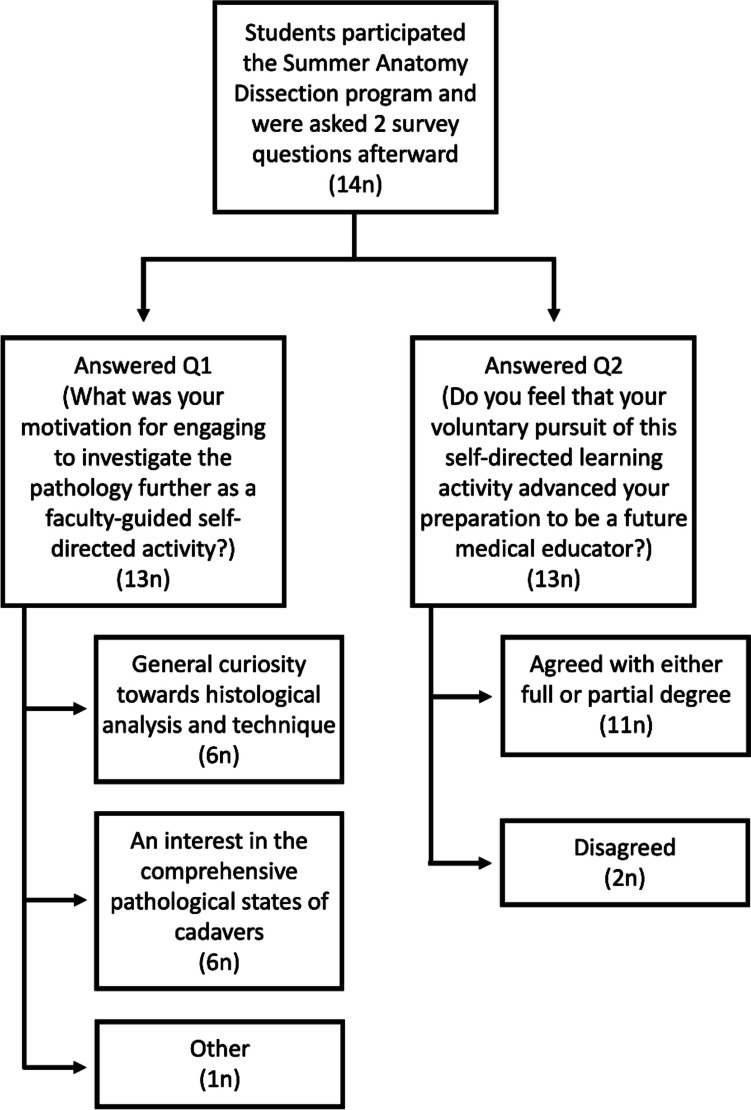
Students who participated in the summer Dissection Program and the SDL were surveyed regarding their educational experiences. Their responses are tabulated here after categorization of the themes

Free form text responses to the two questions were gathered, granting insight into student experiences. In total, 13 of the 14 students answered the survey. Qualitative analysis of the responses was used to identify the themes. Two sets of investigators independently manually identified themes based on an open coding system. They identified frequently occurring concepts to create key words. The scripts were coded independently and compiled for a consensus discussion for the final themes. On compilation, five themes emerged. A density analysis was done to determine the relative importance of each theme (Fig. 1).

## Results

### Question 1

Five positive themes were identified as the motivation for engagement. These included (1) educational goals such as pursuit of unique pathology, (2) learning the clinical context and application of knowledge, (3) curiosity, (4) learning histological skills, and (5) research. No negative themes were identified.

While expected themes of analyzing unique pathology and pursuing histology skills were present, unanticipated themes also emerged. Students stated their motivation was inspired by a general curiosity and valued the clinical context provided by the project. Students felt empowered to explore a testable clinical hypothesis. A common theme among the group was the significance of incorporating physical findings and donor records in their histological investigations to cultivate crucial application of knowledge and analytical skills, akin to real-world scenarios. Students believed this experience would allow greater insight and help build their medical reasoning skills. These students believed this extracurricular program provided a unique opportunity to grow this insight as their formal curriculum did not include such methods of teaching and learning.

### Question 2

On qualitative analysis, two positive themes emerged (1) development as a medical educator (2) contextual learning that provided deeper understanding. A minor negative theme was (1) not effective at providing insights into teaching.

In total, 11 students agreed that this program fully or partially prepared them to become future medical educators. Students appreciated the focus on the holistic view of the human body and its pathologies, its relation to clinical education, and acquisition of technical skills. Among these students, 3 students mentioned explicitly that the acquisition of knowledge and practical skills as benefiting their preparation to becoming a future medical educator. Similarly, 1 student described how increased teamwork would aid in this aim.

## Discussion

Overall, most students (85%) identified the potential of cadaveric dissection and the associated SDL activity for assisting their development as future medical educators. Students offered various reasons for their motivation to explore their findings. For example, some participants identified an intrinsic motivation to test their hypothesis against the provided cause of death. With support from their instructors as resources, students were able to develop their proficiency in communication and clinical reasoning skills. Participants were interested in acquiring technical skills, where one student stated that “Understanding how tissues are sectioned and prepared was extremely rewarding.” Others desired to engage in collaboration with their peers and faculty. Students identified the value of cooperation and support. One student response explained, “when trying to do something unfamiliar, it is really helpful to have people around you that you can collaborate with and ask questions to.”

Multiple students hoped to utilize the SDL activity as a bridge between their medical education and their future roles as physicians, with one student writing that “Having the opportunity to look at the pathology at a deeper level and understand how it might have affected the patient and their signs, symptoms, and cause of death was a way to broaden my understanding in a hands-on learning opportunity. Additionally, it allowed me to be curious.” Similarly, another student reported that they had “…never seen those kinds of pathology grossly and in person, and I was interested in learning more about it and investigating it more closely. Furthermore, I also felt that by investigating pathology more, it would benefit my learning both as a medical student and future physician. It provided a unique learning opportunity to see how a pathology looks, in the context of a body, and potential consequences. I hoped that it'd help me learn the material better.”

Student-centered SDL activity in the context of anatomy dissection offers the opportunity of promoting a relationship between the basic sciences and clinical concepts [4, 9]. It is believed that this bridging can aid students’ motivation in pursuing the role of medical educators, fostering their technical proficiency, medical understanding, and general curiosity. In this additional training program, students were supported in becoming “visually literate” and encouraged to partake in the preparation and identification of tissues, where “it is rare…to see an isolated student hunched over a compound microscope with a box full of glass slides and textbook open,” let alone identifying their own resected tissues [10]. It is believed that in further focusing on student cooperation, in a faculty-supported setting, such a program can function to encourage the development of skills related to the production of medical educators.

The results demonstrate that students have diverse motivations for engaging in such programs, including intrinsic curiosity, acquisition of technical skills, group work, and preparation for their future roles as physicians and medical educators. By focusing on these aspects, a learning environment aiming to encourage application of knowledge and contextual clinical application could be designed. A formalized program modeled after the voluntary SDL activities developed by these dissection lab students would benefit students’ skills in developing their own in-depth knowledge and practical skills via SDL opportunities. This course would employ the findings related to the values of cooperation, curiosity, and the supported acquisition of application of knowledge and technical skills. SDL activities would be useful to enhance their comprehension of the subject while also developing their clinical contextual skills and understanding of pathophysiology. While these could be strengths of the program, they could also be limitations of the program as they depend on the interest and level of student engagement.
